# Two Cases of Knee Dislocation With Multiligament Ruptures Caused by Rotary Tiller Injury

**DOI:** 10.1002/ccr3.72507

**Published:** 2026-04-17

**Authors:** Fuhang Shuang, Chengxiong Li, Jianbang Hu, Huaixu Song, Qingsong Shi, Genbi Jiang, Zhengxiong Zhang, Jin Jia

**Affiliations:** ^1^ Department of Traumatic Surgery Northeast Yunnan Central Hospital Zhaotong China

**Keywords:** knee dislocation, ligament reconstruction, multiligament rupture, rotary tiller injury

## Abstract

Rotatory knee dislocation resulting from rototiller injury is an uncommon clinical entity, typically accompanied by multiligamentous (Anterior Cruciate Ligament [ACL], Posterior Cruciate Ligament [PCL], Medial Collateral Ligament [MCL], Lateral Collateral Ligament [LCL]) and meniscal injuries. This report details the diagnostic approach, therapeutic management, and postoperative rehabilitation of two patients who sustained knee dislocations with multiple ligament ruptures due to rototiller accidents. It underscores the critical importance of prompt closed or open reduction alongside immediate assessment for potential vascular and neurological compromise. Following exclusion of such injuries, one‐stage multiligament reconstruction and meniscal repair are recommended within three weeks. Surgical intervention performed promptly after edema resolution, complemented by early postoperative functional exercise, can lead to favorable knee functional outcomes.

## Key Clinical Message

Rototiller‐induced knee dislocation often involves multiligament/meniscal injuries. Urgent neurovascular assessment, prompt reduction, one‐stage reconstruction within 3 weeks, and early rehabilitation are crucial for favorable functional outcomes.

## 1 Introduction

The mechanization of agriculture has led to widespread use of rotary tillers in Southwest China, resulting in an increasing incidence of related injuries. Rotary tiller injuries typically involve lacerations, crush injuries, avulsions, and even amputations. However, knee dislocation caused by such injuries is uncommon. This report presents two cases of knee dislocation with multiligament ruptures resulting from rotary tiller accidents. The first case involved a particularly complex rotational dislocation pattern. We share these cases to provide clinical insights into the management of this specific injury mechanism, emphasizing the importance of comprehensive neurovascular evaluation and timely surgical intervention.

Knee dislocation represents a severe trauma often associated with vascular and nerve injuries, and disruption of multiple ligamentous structures [1]. Epidemiological data indicates an annual incidence of approximately 15.3 per million, with young males (18–29 years) at highest risk [2]. High‐energy mechanisms like this pose a significant threat to the popliteal artery due to compression, stretching, or laceration; missed diagnosis can lead to limb ischemia, necrosis, and amputation [3]. Although vascular injury occurs in about 3% of peri‐knee fractures, its incidence rises to roughly 16% in traumatic knee dislocations [4]. Screening relies on the clinical triad (absent dorsalis pedis pulse, cool limb, capillary refill > 2 s) and ankle‐brachial index (ABI) < 0.9 warrants urgent CTA or arteriography [1]. Both cases reported here underwent thorough vascular assessment including palpable dorsalis pedis pulses, normal temperature, adequate capillary refill, and ABI measurements > 0.9. As all these parameters were normal, no further advanced imaging (such as CTA) was deemed necessary, and the patients were managed with close clinical observation of limb perfusion. This approach aligns with current guidelines that recommend CTA only when physical examination or ABI is abnormal.

## 2 Case Report

### 2.1 Case History/Examinations

#### 2.1.1 Case 1

**FIGURE 1:**
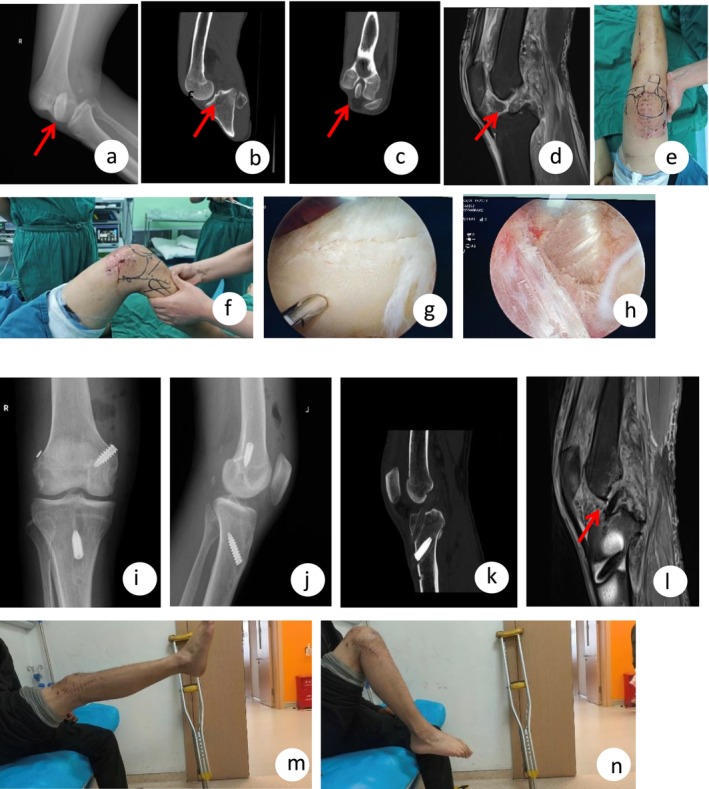
Case 1—Imaging and intraoperative findings of a case with right knee rotatory dislocation. (a) Anteroposterior radiograph at admission demonstrating rotatory dislocation of the right knee. (b, c) Three‐dimensional CT reconstructions on admission confirming knee dislocation with the patella displaced and incarcerated within the femoral intercondylar notch. (d) Post‐reduction MRI following emergency open reduction, revealing ruptures of the anterior and posterior cruciate ligaments. (e–h) Preoperative examination under anesthesia and intraoperative arthroscopic views during multiligament reconstruction performed two weeks after admission. (i–l) Postoperative anteroposterior and lateral radiographs along with CT images of the right knee demonstrating satisfactory positioning of suture anchors and reconstructed ligament tunnels. (h) MRI of the reconstructed anterior and posterior cruciate ligaments following multiligament reconstruction, showing restored ligament integrity. (m, n) Follow‐up photographs at one month postoperatively, showing active knee extension and flexion achieved by the patient.

A 47‐year‐old male presented to the emergency department 2 h after a rotary tiller injury, complaining of severe right knee pain and inability to move the joint. The right knee was held in flexion, valgus, and external rotation. Mild anterior skin abrasion was noted without open wounds or bleeding. Significant swelling was present. Diffuse tenderness was elicited, and the patella was non‐palpable. Active and passive range of motion was severely restricted. The right leg was shortened by 2 cm. Provocative tests (anterior/posterior drawer, varus/valgus stress, Lachman) were impossible due to pain. Neurovascular examination revealed a palpable dorsalis pedis pulse, intact sensation in all dermatomes, and normal capillary refill (< 2 s). Ankle‐brachial index (ABI) was 1.0 on the affected side. Given the normal findings, no CTA was performed, and the patient was monitored closely for any signs of vascular compromise. Radiographs and CT scans confirmed a rotational dislocation of the right knee with femoral condyle valgus and external rotation. The patella was dislocated laterally and incarcerated within the femoral intercondylar notch (Figure 1a–c).

#### 2.1.2 Case 2

**FIGURE 2:**
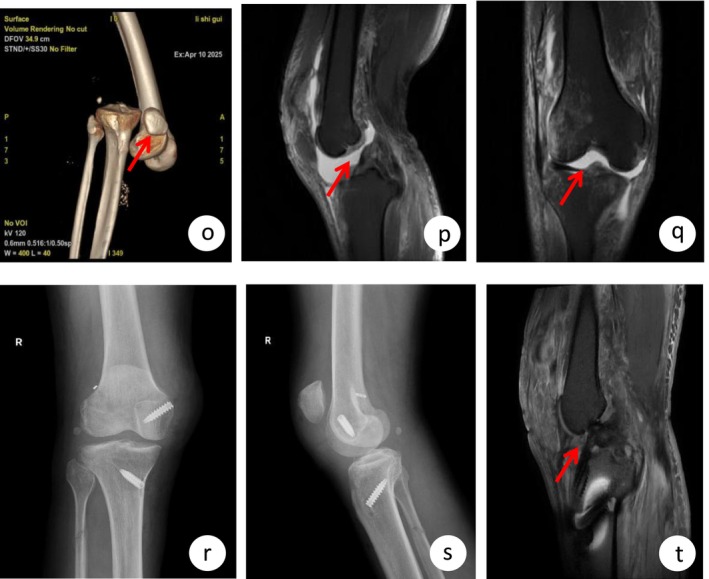
Case 2—Pre‐ and postoperative imaging of a case with traumatic right knee dislocation. (o) Three‐dimensional computed tomography (3D CT) obtained at admission clearly illustrates the dislocated knee joint. (p, q) Magnetic resonance images (MRI) performed after emergent surgical reduction show complete tears of both the anterior and posterior cruciate ligaments. (r, s) Postoperative anteroposterior and lateral knee radiographs document the positioning of internal fixation following multiligament reconstruction surgery. (t) A follow‐up MRI study visualizes the reconstructed cruciate ligaments and confirms their anatomic alignment and integrity.

A 49‐year‐old male presented 2 h after a rotary tiller injury, with right knee pain and bleeding. The knee was in flexion deformity with mild external rotation and valgus. Three 5‐6 cm open wounds were present anteriorly and mediolaterally over the knee. Significant tenderness was present. Sensory examination was normal, dorsalis pedis pulse was palpable, and capillary refill was < 2 s. ABI measured 0.95. As these findings were unremarkable, CTA was not pursued, and the patient was kept under vigilant observation for any change in vascular status. Active/passive motion was severely restricted. Peripheral circulation was adequate. Radiographs and CT confirmed posterior dislocation of the tibia and lateral dislocation of the patella (Figure 2o).

### 2.2 Investigations and Treatment

#### 2.2.1 Case 1

Emergency open reduction under epidural anesthesia was performed via a 10‐cm anterior midline incision. Findings included quadriceps muscle injury/partial tear, joint dislocation (tibia internally rotated, femur externally rotated), patellar dislocation, quadriceps tendon separation, joint capsule rupture, anterior cruciate ligament (ACL) rupture, posterior cruciate ligament (PCL) injury, and medial collateral ligament (MCL)/lateral collateral ligament (LCL) ruptures. The incarcerated patella confirmed on pre‐op imaging was visualized. Significant tension in the quadriceps mechanism, trapped against the tibial plateau, made reduction difficult. A 4‐cm lateral femoral incision facilitated traction and successful reduction. The knee was stabilized in a plaster cast post‐reduction. Low‐molecular‐weight heparin (LMWH, 5000 IU once daily) was administered for 7 days postoperatively, after which it was switched to rivaroxaban (10 mg once daily) for an additional 3 weeks. Follow‐up Doppler ultrasound showed no evidence of deep vein thrombosis. MRI performed 4 days post‐reduction confirmed ruptures of the ACL, PCL, MCL, LCL, a tear in the posterior horn of the lateral meniscus, and partial injuries to the patellar tendon and medial patellofemoral ligament (Figure 1d). At 2 weeks, after incision healing, definitive surgery was performed: Arthroscopic ACL/PCL reconstruction, MCL/LCL repair, and lateral meniscus suture. The PCL was reconstructed using an artificial tendon. The ACL was reconstructed using autologous semitendinosus, gracilis, and half of the peroneus longus tendons (woven to 8 mm diameter). Autografts were chosen over allografts primarily due to the patients' relatively young age and limited financial resources, aiming to reduce medical costs while ensuring reliable graft healing. Additionally, allografts were not readily available at our institution at the time of surgery. The lateral meniscus tear (red‐red zone) was repaired with two sutures. ACL/PCL tibial and femoral tunnels (8 mm) were created using guides. The PCL was reconstructed using an artificial tendon (braided polyetheretherketone (PEEK) reinforced tendon, 8 mm diameter, 120 mm length, selected for its excellent biomechanical strength with tensile strength ≥ 2500 N), fixed with interference screws on the femoral and tibial sides. The ACL graft (autologous) was fixed with an adjustable suspensory fixation device on the femur and an interference screw on the tibia. The MCL (torn from its femoral attachment) was repaired using a suture anchor and modified Mason‐Allen technique. The LCL was repaired similarly (Figure 1e–h). Postoperative follow‐up examinations revealed restored anatomical alignment of the knee joint and satisfactory reconstruction of the injured ligaments (Figure 1i–l).

#### 2.2.2 Case 2

Emergency debridement of the open injury and open reduction under epidural anesthesia were performed, followed by plaster cast immobilization. Cefuroxime was administered. Low‐molecular‐weight heparin (LMWH, 5000 IU once daily) was given for 7 days, then switched to rivaroxaban 10 mg daily for 3 weeks. No thrombotic events were detected on follow‐up. Non‐weight‐bearing was instructed. MRI performed 4 days post‐reduction (after swelling subsided) confirmed ruptures of the ACL, PCL, MCL, LCL, and a tear in the posterior horn of the lateral meniscus (Figure 2p,q). Due to wound necrosis and three episodes of re‐dislocation triggered by alcohol withdrawal hysteria, definitive surgery was delayed until 4 weeks post‐injury. The procedure mirrored Case 1: Arthroscopic ACL reconstruction (using autologous semitendinosus and gracilis), PCL reconstruction using artificial tendon (PEEK artificial tendon), and given the lateral patellar dislocation noted on initial presentation, medial patellofemoral ligament (MPFL) reconstruction was performed using gracilis autograft, and the lateral retinaculum was repaired. MCL repair with anchor and lateral meniscus suture were also performed. Postoperative follow‐up examinations revealed restored anatomical alignment of the knee joint and satisfactory reconstruction of the injured ligaments (Figure 2r–t).

## 3 Postoperative Rehabilitation

Both cases received structured rehabilitation under the supervision of a physical therapist, tailored to their surgical timing and soft tissue conditions. Case 1: Postoperatively, the knee was immobilized in a hinged brace (0°–30° flexion) for 2 days. On postoperative day 3, passive knee flexion (PROM) was initiated (target: 60° at 1 week, 90° at 2 weeks). Active knee flexion/extension (AROM) training started at 2 weeks postoperatively, with proprioception training added at 4 weeks. Partial weight‐bearing (50% body weight) was allowed at 3 weeks, and full weight‐bearing was achieved at 6 weeks. Return to light daily activities was permitted at 3 months. Case 2: Due to delayed surgery (4 weeks post‐injury) and wound healing issues, rehabilitation was modified: Immobilization in a hinged brace (0°–20°) for 3 days, PROM initiated at postoperative day 4 (target: 50° at 1 week, 80° at 3 weeks), AROM at 3 weeks, and partial weight‐bearing at 4 weeks. Full weight‐bearing was achieved at 8 weeks, with functional training adjusted to avoid soft tissue overload.

### 3.1 Conclusion and Results

#### 3.1.1 Case 1

At 1‐month follow‐up, the incisions were well‐healed. The patient was ambulating with crutches. Knee range of motion (ROM) was 0°–110° (Figure 1m,n). Anterior/posterior drawer and varus/valgus stress tests were negative. HSS score was 92. At 6‐month follow‐up, ROM improved to 0°–135°, the patient was walking independently with a normal gait, knee stability was excellent, and HSS (Hospital for Special Surgery) score was 98.

#### 3.1.2 Case 2

The patient was highly satisfied. At 1‐month follow‐up, the incisions were well‐healed. The patient was ambulating with crutches. Knee ROM was 0°–80°. Anterior/posterior drawer and varus/valgus stress tests were negative. HSS score was 80.

## 4 Discussion

Rotary tiller injuries causing knee dislocation are rare, constituting a subset of traumatic knee dislocations. As highlighted by the Finnish registry and others, the paramount concern in any knee dislocation is neurovascular injury, particularly to the popliteal artery [3, 4]. A meticulous vascular examination (pulses, temperature, capillary refill) and ABI measurement are essential first steps [1]. In both presented cases, comprehensive neurovascular assessment including physical examination and ABI yielded normal results. According to current guidelines, advanced imaging such as CTA is indicated only when the physical examination or ABI is abnormal (< 0.9). Therefore, we opted for close clinical monitoring without CTA, and neither patient developed any vascular complications. This approach underscores that in the absence of hard signs or ABI abnormality, careful observation may suffice, although vigilance remains paramount.

Prompt reduction is critical to alleviate pain, potentially reduce further neurovascular compromise, and facilitate subsequent management [5]. While closed reduction is sometimes possible (35.5% in one study [2]), Case 1 required open reduction due to the complex rotational deformity and patellar incarceration within the intercondylar notch, blocking reduction. Case 2 required open reduction initially due to the open injury.

Traumatic knee dislocation almost invariably involves multiligament injury (≥ 2 of ACL, PCL, MCL, LCL), reported in up to 95% of cases [6]. Post‐reduction clinical stability assessment and MRI are crucial for definitive diagnosis and planning [7]. Current evidence strongly favors surgical reconstruction or repair of multiligament injuries over non‐operative management for better functional outcomes [8, 9]. The timing of surgery is also critical. Early reconstruction (≤ 3 weeks) is generally associated with superior functional results and reduced stiffness compared to delayed procedures (> 3 weeks) [10, 11]. This principle is starkly illustrated by our cases: Case 1, reconstructed at 2 weeks, achieved near‐normal function (HSS 92) by 6 months. Case 2, delayed to 4 weeks due to complications (wound issues, re‐dislocation), had significantly lower function (HSS 80) at 1 month, although longer follow‐up might show further improvement. Both cases also involved lateral meniscus tears, underscoring the need for thorough intra‐articular assessment during reconstruction.

In Case 2, the presence of lateral patellar dislocation with MPFL/LPFL involvement required additional attention. MPFL reconstruction using gracilis autograft and lateral retinaculum repair was performed to address patellofemoral instability, which is often overlooked in multiligament knee injuries but critical for comprehensive functional recovery.

Graft choice (autograft vs. allograft vs. artificial tendon) remains debated, with no clear consensus on superiority for functional outcomes in multiligament reconstruction. In our cases, autografts were selected over allografts primarily because the patients were relatively young and had limited financial resources. Autografts offer reliable biological healing without the added cost of allografts, making them a cost‐effective choice in our setting. Although allografts may reduce operative time and avoid donor‐site morbidity, the economic burden on patients in our region is a significant consideration. The primary surgical goal remains achieving anatomical reconstruction and robust stability, regardless of graft type.

Post‐operative rehabilitation is another cornerstone of success. Although the optimal timing to initiate motion is debated, early controlled mobilization (as practiced in Case 1 starting at 2 days post‐op, aiming for 0°–110°) is widely recommended to prevent arthrofibrosis and optimize functional recovery.

Rotary tiller injuries, while typically causing limb lacerations or crush injuries, can rarely result in devastating knee dislocations with multiligament ruptures. Immediate priorities include thorough neurovascular assessment and prompt joint reduction (open or closed). Definitive management involves surgical reconstruction or repair of the ruptured ligaments and associated meniscal/cartilage injuries. Arthroscopic‐assisted open knee surgery serves as the optimal approach for these complex injuries, as it allows precise visualization of intra‐articular structures (e.g., meniscal tears, ligament stumps) to guide anatomical reconstruction, minimizes soft tissue trauma compared to traditional open surgery, and reduces the risk of postoperative arthrofibrosis—key factors for functional recovery in multiligament injured knees. Both cases were managed with a single‐stage surgical approach: After emergency reduction and stabilization, definitive treatment (multiligament reconstruction, collateral ligament repair, and meniscal suture) was performed in one operation once soft tissue conditions improved (swelling resolution, wound healing). Single‐stage surgery was preferred to minimize surgical trauma, avoid repeated joint manipulation, and optimize ligament healing by reducing the interval between injury and reconstruction—consistent with current evidence supporting single‐stage intervention for multiligament knee injuries when soft tissue and patient conditions permit. Early surgery (≤ 3 weeks post‐injury), when feasible, combined with a structured rehabilitation program emphasizing early motion, leads to significantly better functional outcomes, as demonstrated by the superior results in Case 1 compared to the delayed Case 2. Vigilance for complications like wound problems and patient comorbidities is essential.

## Author Contributions

**Fuhang Shuang:** writing – review and editing. **Chengxiong Li:** resources, writing – review and editing. **Jianbang Hu:** writing – review and editing. **Huaixu Song:** data curation. **Qingsong Shi:** supervision. **Genbi Jiang:** investigation. **Zhengxiong Zhang:** supervision. **Jin Jia:** software.

## Funding

Effect of High Tibial Osteotomy on Ankle Joint Stress and Three‐Dimensional Finite Element Analysis was funded by Yunnan Provincial Department of Education, China. Construction of Machine Learning Thrombosis Prediction Model After Joint Replacement Based on Ultrasonic Hemodynamic Indicators was funded Northeast Yunnan Central Hospital. This work was supported by Construction of Machine Learning Thrombosis Prediction Model After Joint Replacement Based on Ultrasonic Hemodynamic Indicators, the Yunnan Provincial Department of Education Teac Effect of High Tibial Osteotomy on Ankle Joint Stress and Three‐Dimensional Finite Element Analysis, Yunnan Provincial Department of Education Teacher.

## Consent

Written informed consent was obtained from the patients for publication of this case report and accompanying images.

## Conflicts of Interest

The authors declare no conflicts of interest.

## Supporting information


**Data S1:** Supporting Information.

## Data Availability

The data that support the findings of this study are available from the corresponding author upon reasonable request.
