# Supplementary material for: Two Cases of Knee Dislocation With Multiligament Ruptures Caused by Rotary Tiller Injury
Source: Clin Case Rep. 2026 Apr 17;14(4):e72507. doi: 10.1002/ccr3.72507 (PMC13090157; doi:10.1002/ccr3.72507)
Supplement: Supplementary file 1 — Data S1: Supporting Information. [file CCR3-14-e72507-s001.pdf]

Fund Project: Effect of High Tibial Osteotomy on Ankle Joint Stress and Three-Dimensional Finite Element Analysis，Supported by the Yunnan Provincial Department of Education Teacher Research Grant (2025J0352)

Construction of Machine Learning Thrombosis Prediction Model After Joint Replacement Based on Ultrasonic Hemodynamic Indicators，Supported by the Yunnan Provincial Department of Education Teacher Research Grant (2025J0352) and clinical research funding from the Diandongbei Central Hospital of Yunnan Province (2025ZXYY008).
